# Prevalence and incidence of prediabetes in Latin America. A systematic review and meta-analysis

**DOI:** 10.1007/s40200-024-01549-6

**Published:** 2024-12-27

**Authors:** Víctor Juan Vera-Ponce, Joan A. Loayza-Castro, Fiorella E. Zuzunaga-Montoya, Nataly Mayely Sanchez-Tamay, Juan Carlos Bustamante-Rodríguez, Lupita Ana Maria Valladolid-Sandoval, Luisa Erika Milagros Vásquez-Romero, Carmen Inés Gutiérrez de Carrillo

**Affiliations:** 1 Instituto de Investigación de Enfermedades Tropicales, Universidad Nacional Toribio Rodríguez de Mendoza de Amazonas (UNTRM), Amazonas, Perú; 2 Facultad de Medicina (FAMED), Universidad Nacional Toribio Rodríguez de Mendoza de Amazonas (UNTRM), Amazonas, Perú; 3 Universidad Continental Lima, Lima, Perú

**Keywords:** Prediabetic state, Prevalence, Incidence, Systematic review, Meta-analysis, Latin America (source: MeSH NLM)

## Abstract

**Introduction:**

Prediabetes represents a significant public health challenge in Latin America. Its prevalence varies considerably depending on the diagnostic criteria used, which hinders a precise understanding of its magnitude in the region.

**Objective:**

To estimate the prevalence and incidence of prediabetes in Latin America through a systematic review (SR).

**Methods:**

A SR and meta-analysis was conducted searching through October 25, 2024 in SCOPUS, EMBASE, Web of Science, and PubMed. Studies were included if they: (1) used probabilistic sampling methods, (2) included adult participants (≥ 18 years), (3) assessed prediabetes using WHO criteria, fasting glucose, postprandial glucose, or HbA1c, and (4) were published in English, Spanish, or Portuguese. Studies using non-probabilistic sampling, focusing on specific medical conditions, or lacking sufficient data to calculate prevalence or incidence were excluded. Random-effect models were used to estimate pooled prevalence, with heterogeneity assessed using I² statistics and publication bias through funnel plots.

**Results:**

Twenty-five studies from 9 countries published between 1992 and 2023 were analyzed. The pooled prevalence of prediabetes was 24% (95% CI: 18–30%). According to specific criteria, the prevalences were: WHO 11% (95% CI: 5–18%), FG 18% (95% CI: 10–27%), PPG 20% (95% CI: 3–46%), and HbA1c 32% (95% CI: 21–52%). High heterogeneity was observed among studies (I² = 99–100%, *p* < 0.001). Only one study analyzed the incidence, which was 12.8%.

**Conclusions:**

Prediabetes prevalence in Latin America is high, with significant variations by diagnostic criteria. The limited number of incidence studies and high heterogeneity highlight the need for standardized approaches in future research. Implementation of preventive strategies and strengthening of epidemiological surveillance systems are crucial for addressing this public health challenge.

**Supplementary Information:**

The online version contains supplementary material available at 10.1007/s40200-024-01549-6.

## Highlights

- The systematic review included studies published between 1992 and 2023, spanning more than three decades of research on prediabetes in nine Latin American countries: Argentina, Brazil, Colombia, Costa Rica, Dominican Republic, Ecuador, Mexico, Peru, and Venezuela
- While some recent studies report higher prevalences (e.g., Souza Marques dos Santos 2020 and Lazo-Porras 2020 reporting >30%) compared to earlier studies (e.g., Pablo Aschner 1992 and Bosi 2009 reporting <10%), these differences cannot be interpreted as direct evidence of temporal trends
- Our meta-analysis revealed substantial variations: WHO criteria showed the lowest prevalence at 11% (95% CI: 5-18%), followed by fasting glucose at 18% (95% CI: 10-27%), postprandial glucose at 20% (95% CI: 3-46%), and HbA1c showing the highest at 32% (95% CI: 21-52%).
- Factors such as urbanization, changes in dietary patterns, sedentarism, and socioeconomic disparities, which vary significantly within the region, likely contribute to these differences and should be considered in the design of interventions.

**Supplementary Information:**

The online version contains supplementary material available at 10.1007/s40200-024-01549-6.

## Introduction

Prediabetes, defined as an intermediate state of hyperglycemia where blood glucose levels are above normal but below the diagnostic threshold for type 2 diabetes mellitus (T2DM), has become a significant global public health concern [1]. This condition not only substantially increases the risk of developing T2DM but is also associated with a higher risk of cardiovascular diseases and microvascular complications [2].

The global burden of prediabetes varies significantly across regions and populations. A recent systematic review and meta-analysis by the International Diabetes Federation estimated that the worldwide prevalence of impaired glucose tolerance in 2021 was 9.1% ^(3)^. However, this global estimate faces essential limitations when applied to Latin America. Notably, in this international review, Latin America was represented by data from only one country (Bolivia), which cannot adequately describe the diversity of the region’s population, healthcare systems, and socioeconomic conditions. This limited representation underscores a significant gap in our understanding of prediabetes epidemiology in Latin America, where factors such as rapid urbanization, dietary transitions, and varying healthcare access patterns may contribute to distinct prevalence patterns. The lack of comprehensive regional data hampers the development of targeted public health strategies and resource allocation for prediabetes prevention and management in Latin American populations.

In Latin America, the burden of diabetes and its precursor conditions has increased significantly in recent decades, driven by lifestyle changes, urbanization, and population aging [4]. The region is experiencing an accelerated epidemiological and nutritional transition characterized by increasing obesity and sedentary behavior, factors closely related to the development of prediabetes and diabetes [5]. However, the exact magnitude of the prediabetes problem in Latin America is not well established, with estimates varying widely among countries and subpopulations.

Understanding the prevalence and incidence of prediabetes is crucial for planning public health strategies and allocating resources in the region. Early identification and management of individuals with prediabetes offer a unique opportunity to prevent or delay the development of T2DM and its associated complications [6]. Lifestyle interventions and, in some cases, pharmacological interventions effectively prevent the progression from prediabetes to diabetes [7]. However, to implement these strategies effectively, it is essential to have accurate data on the magnitude of the problem in different Latin American contexts.

In this context, this systematic review focused exclusively on studies using probabilistic sampling to estimate the prevalence and incidence of prediabetes in Latin America. This approach aims to provide a more accurate and generalizable synthesis of available evidence, minimizing potential biases associated with non-probabilistic sampling methods.

## Methodology

### Design

A systematic review (SR) with meta-analysis of cross-sectional or longitudinal studies from Latin America was conducted, following the guidelines of the PRISMA statement (Preferred Reporting Items for Systematic Reviews and Meta-Analyses) [8], adapted for an SR of prevalence and incidence studies.

### Search strategy

A strategic search was conducted in six academic databases, Scopus, Web of Science, Embase, and PubMed, from the inception of each database until October 18, 2024. The key terms used for the search in all sources were “prevalence,” “incidence,” “prediabetes,” “pre-diabetes,” “impaired fasting glucose,” “impaired glucose tolerance,” and “Latin America,” or the names of specific Latin American countries.

The search strategy was adapted for each database, using a controlled vocabulary (such as MeSH terms in PubMed) when available, combined with free text search. Publications in English, Spanish, and Portuguese were included. No restrictions were applied regarding the year of publication to capture all relevant available studies.

In addition to electronic databases, manual searches were conducted in the reference lists of included articles and relevant previous systematic reviews to identify additional studies that might have been missed in the electronic search.

The detailed search strategy for each database is provided in Supplementary Appendix 1.

### Selection criteria

Studies were eligible for inclusion if they met several specific criteria. First, observational studies published as full articles were considered, including cross-sectional studies for prevalence data and longitudinal studies for incidence data. The study population had to include adult participants (≥ 18 years) of both sexes residing in Latin American countries.

Regarding prediabetes measurement, studies had to assess prevalence or incidence using at least one of the following blood markers: fasting glucose (FG), oral glucose tolerance test (OGTT), or glycated hemoglobin (HbA1c). These markers had to follow the diagnostic criteria recommended by the American Diabetes Association (ADA) or the World Health Organization. According to the ADA criteria [9], prediabetes is diagnosed when fasting glucose levels are between 100 and 125 mg/dL (5.6–6.9 mmol/L), or when 2-hour postprandial glucose during a 75 g oral glucose tolerance test (OGTT) ranges from 140 to 199 mg/dL (7.8–11.0 mmol/L), or when glycated hemoglobin (HbA1c) values fall between 5.7 and 6.4% (39–47 mmol/mol).

The WHO criteria differ slightly, particularly in fasting glucose and HbA1c thresholds. WHO defines prediabetes as fasting glucose levels between 110 and 125 mg/dL (6.1–6.9 mmol/L), 2-hour postprandial glucose during 75 g OGTT between 140 and 199 mg/dL (7.8–11.0 mmol/L), or HbA1c between 6.0 and 6.4% (42–47 mmol/mol). Studies using any one or combination of these criteria were eligible for inclusion. When studies reported prevalences using multiple criteria, we extracted data for each criterion separately to enable subgroup analyses [10].

Additionally, studies had to provide sufficient data to calculate the prevalence or incidence of prediabetes or directly report these measures with their respective confidence intervals. As mentioned earlier, only studies published in English, Spanish, or Portuguese were considered to ensure broad coverage of relevant literature in the region.

On the other hand, exclusion criteria were established to maintain the quality and relevance of the review. Narrative reviews, editorials, letters to the editor, comments, and case studies were excluded, as well as studies using non-probabilistic sampling methods. Studies focusing exclusively on populations with specific medical conditions, such as hospitalized patients or people with pre-existing cardiovascular diseases, were also discarded.

Likewise, studies that did not provide original data, such as predictive models based on secondary data or preclinical or animal studies, were not included. Finally, studies irrelevant to this systematic review’s objective on prediabetes in Latin America were excluded.

### Study selection

For the manage and store articles identified in each explored database, Rayyan software (https://rayyan.qcri.org) was used. This software facilitated the organization and tracking of studies throughout the selection process. Two independent reviewers initially reviewed the titles and abstracts of the identified manuscripts. This first screening phase was conducted anonymously to minimize bias. When both reviewers agreed that a source met the inclusion criteria, it was selected for a more detailed review. In case of a discrepancy between the reviewers, a third reviewer acted as a judge to resolve the disagreement.

Subsequently, a comprehensive review of the full text of all pre-selected articles was conducted. Two reviewers also carried out this process independently, following the same protocol as the initial phase. An Excel spreadsheet recorded the decision to include or exclude each source and specific reasons for exclusion when applicable. Any discrepancy at this stage was resolved through discussion between the reviewers, and if consensus could not be reached, a fourth reviewer was consulted for the final decision.

To ensure a comprehensive selection and minimize the risk of omitting relevant studies, an additional manual search was conducted in the reference lists of included articles and previous systematic reviews on related topics. Studies identified through this method underwent the same selection process described above.

If multiple publications were found based on the same study population, the most recent article or the one providing more complete information on the prevalence or incidence of prediabetes was included.

The entire study selection process was meticulously documented, including the number of studies identified, screened, assessed for eligibility, and included in the review, along with reasons for exclusion at each stage. This information was subsequently presented in a PRISMA flow diagram to provide a clear and transparent view of the selection process.

### Data extraction and qualitative analysis

For each selected study, meticulous data extraction was performed using a Microsoft Excel 2016 template explicitly designed for this systematic review. Two independent reviewers carried out this process to ensure the accuracy and completeness of the extracted data. The template was previously piloted with a sample of included studies to ensure its adequacy and make necessary adjustments before complete extraction.

The extracted data included detailed information on study characteristics and their results. Specifically, the following elements were collected: author(s), year of publication, Latin American country or countries involved, study design, data collection period, sample size, demographic characteristics of the studied population (such as age and sex), probabilistic sampling method used, diagnostic criteria employed for prediabetes, blood markers used (fasting glucose, glucose tolerance test, or glycated hemoglobin), reported prevalence or incidence of prediabetes, and measures of variability (such as 95% confidence intervals or standard errors).

A narrative synthesis of included studies was performed for qualitative analysis, highlighting general trends in the prevalence and incidence of prediabetes in Latin America and variations observed between countries, regions, or population subgroups. Special attention was paid to the consistency of definitions and diagnostic criteria used for prediabetes across different studies, and the implications of any observed heterogeneity in these aspects were discussed.

Discrepancies in data extraction were resolved following the same consensus process described in the study selection section.

### Risk of bias assessment

Two of our researchers independently assessed the risk of bias for all studies in the systematic review. For this purpose, the bias tool by Munn et al. [11] was used. This tool was selected because it was specifically developed to increase consistency in systematic reviews of prevalence data and is widely recommended as the most appropriate tool for studies of this type.

The assessment was based on nine specific established criteria: (1) The appropriateness of the sampling frame for the target population; (2) The proper selection of participants; (3) The adequacy of the sample size; (4) The detailed description of subjects and study context; (5) The conduct of data analysis that adequately covered the identified sample; (6) The use of valid methods to identify the studied condition; (7) The standard and reliable measurement of the condition in all participants; (8) The appropriateness of statistical methods employed; and (9) The adequate management of response rate or its impact if low.

For each criterion, reviewers assigned a response of “Yes,” “No,” “Unclear,” or “Not applicable.” A total score was calculated for each study, awarding one point for each affirmative response. The level of risk of bias was categorized as follows: studies with a score of 0 to 3 points were considered high risk of bias, those with scores of 4 to 6 points were classified as medium risk of bias, and studies with 7 to 9 points were categorized as low risk of bias.

Additionally, when appropriate, publication bias was assessed through visual inspection of funnel plots and applying statistical tests, such as Egger’s test. These assessments were conducted to determine if there was evidence of bias in the literature included in the SR.

### Quantitative analysis

All quantitative analyses were performed using R statistical software version 4.1.2 and the ‘meta’ package version 5.2-0. For the meta-analysis, all studies that provided data on the prevalence of prediabetes, assessed by at least one of the blood markers mentioned above (fasting glucose, oral glucose tolerance test, or glycated hemoglobin), were included. Each study’s total sample size (n) and number of cases (r) were extracted.

The meta-analysis used the ‘metaprop’ function from the ‘meta’ package. The Freeman-Tukey double arcsine transformation method (sm = “PFT”) was used for proportion transformation, which is particularly useful when working with proportions close to 0 or 1. Confidence intervals were calculated using the Clopper-Pearson method (method.ci = “CP”), which provides exact confidence intervals for proportions.

A random-effects model with the DerSimonian and Laird method (method.tau = “DL”) was used to estimate heterogeneity between studies, given that significant variability was expected due to differences in study populations, measurement methods, and other contextual factors of Latin American countries. The Hartung-Knapp correction (hakn = TRUE) was applied to adjust standard errors, which generally provides more conservative and appropriate results when substantial heterogeneity exists.

For publication bias assessment, we employed multiple analytical approaches to distinguish between publication bias and heterogeneity as sources of funnel plot asymmetry.

Meta-analysis results were presented with their respective 95% confidence intervals and visualized using forest plots using the ‘forest’ function from the ‘meta’ package.

### Ethical aspects

This systematic review and meta-analysis were based exclusively on aggregate data from previously published studies and did not involve collecting individual participant data. Therefore, specific ethical approval was not required for this study. However, it was verified that all studies included in the review had obtained corresponding ethical approval from their respective institutional committees. Additionally, copyright was respected, and all sources used in this systematic review were appropriately cited.

## Results

### Eligible studies

**Fig. 1:**
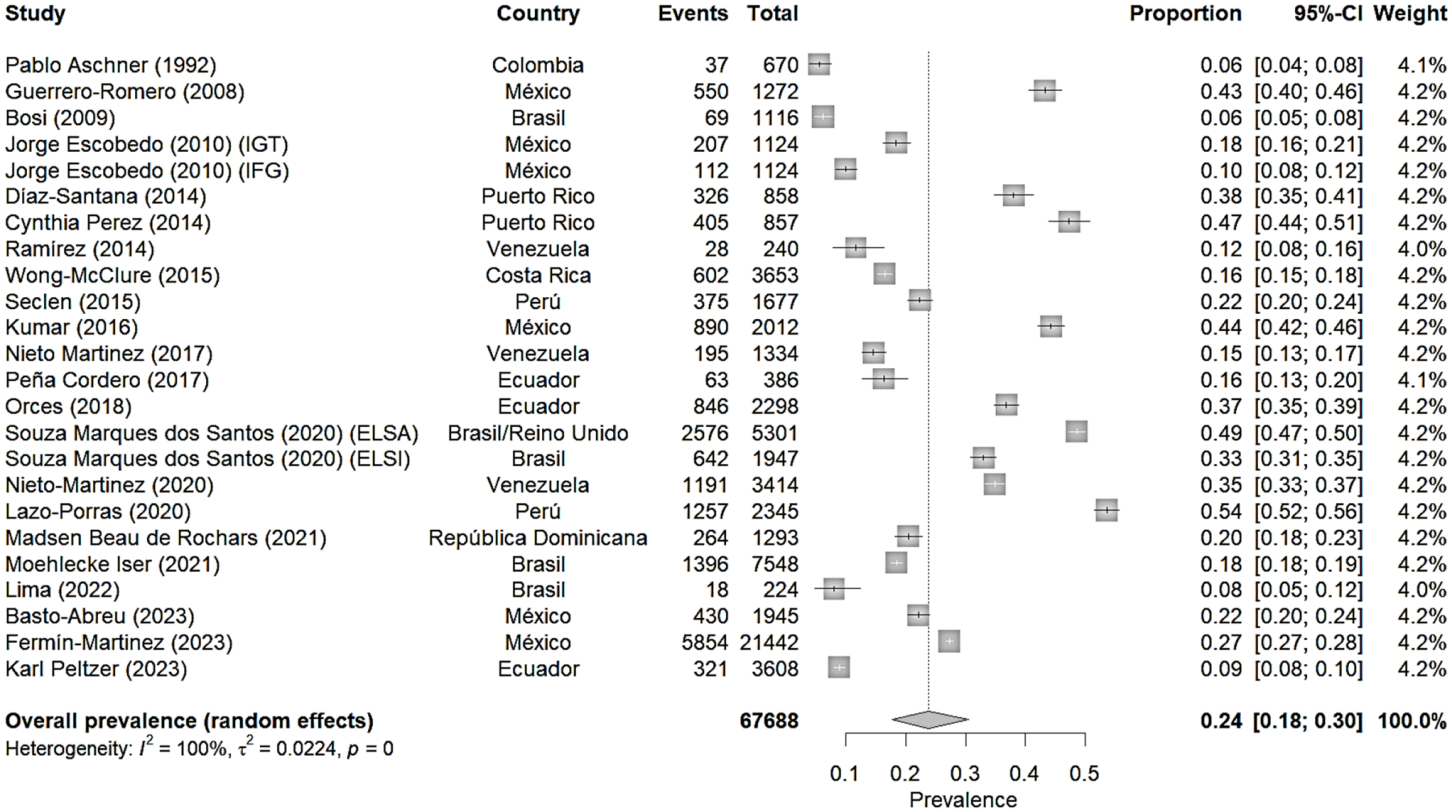
Meta-analysis of prediabetes prevalence according to ADA

A total of 2,198 publications were found. After removing duplicates, 1,178 manuscripts were analyzed based on title and abstract. After excluding 983 studies based on title and abstract screening, 195 full articles were assessed for eligibility. Finally, after applying the selection criteria and excluding 170 full-text articles, 25 articles were selected for the systematic review (see Fig. 1).

### Study characteristics

The systematic review included studies published between 1992 and 2023, spanning more than three decades of research on prediabetes in nine Latin American countries: Argentina, Brazil, Colombia, Costa Rica, Dominican Republic, Ecuador, Mexico, Peru, and Venezuela [12–36]. This temporal breadth allowed for capturing the evolution of prediabetes epidemiology in the region and changes in diagnostic methods and classification criteria.

Regarding study design, most were cross-sectional analyses (22 studies), while three longitudinal studies were included (Seclen 2015, Maciel De Oliveira 2020, and Lazo-Porras 2020); however, only one of them specifically reported the incidence of prediabetes, which Maciel De Oliveira (2020), with a result of 12.8%. A notable characteristic of this review is the inclusion of multiple studies based on national health surveys and pre-existing databases. For example, Mexico’s National Health and Nutrition Survey (ENSANUT) was used in the studies by Basto-Abreu (2023) [34] and Fermín-Martinez (2023) [35]. The Longitudinal Study of Adult Health (ELSA) in Brazil and its Brazilian counterpart, ELSI, were the basis for the study by Souza Marques dos Santos (2020) [28]. On the other hand, the National Cardiovascular Risk Factor Survey of Costa Rica (2010) was analyzed by Wong-McClure (2015) [21]. Meanwhile, the PERUDIAB study in Peru was the data source for Seclen (2015) [22].

The sample sizes of the included studies varied considerably, reflecting the diversity of research designs and resources in the region. The most miniature study, conducted by Lima in 2022 in Brazil, included 224 participants [33]. In contrast, the most extensive study, conducted by Fermín-Martinez in 2023 in Mexico [35], analyzed data from 21,442 individuals. Most studies (20 out of 25) had samples of more than 1,000 participants, with 11 studies exceeding 3,000 participants (Table 1).

**Table 1:** Characteristics of studies on the prevalence and incidence of Prediabetes in Latin America

Author and Year	Country	Study Type	Inclusion Criteria	Exclusion Criteria	Sampling Method	Sample Size	Age (years)	Women (%)	Diagnostic Criteria	Database (Collection Year)
Pablo Aschner, 1992	Colombia	Cross-sectional analytical	Adults > 30 years, residents > 10 years	NR	Clustered, probabilistic	670	> 30	84	GP: 120–180 mg/dL	Primary Study
Suely Gimeno, 1998	Brazil	Cross-sectional analytical	Japanese-Brazilians aged 40–79	NR	Random, probabilistic	647	40–79	NR	WHO: ADA, GP	Primary Study
Torquato, 2003	Brazil	Cross-sectional analytical	30–69 years	Pregnancy	Clustered random, probabilistic	1,473	30–69	66.5	WHO: FPG and OGTT	Primary Study
Guerrero-Romero, 2008	Mexico	Cross-sectional analytical	Adults 30–65 years, IMSS	Prior diabetes diagnosis	Two-stage random, probabilistic	1,272	44.5*	53	ADA: FPG, OGTT, HbA1c	MexiDiab
Bosi, 2009	Brazil	Cross-sectional analytical	30–79 years, non-diabetic	Pregnancy	Multistage stratified, probabilistic	1,116	52.5*	65	ADA: FPG	Primary Study
Jorge Escobedo, 2010	Mexico	Cross-sectional analytical	≥ 35 years, native, Zapotec/Mixe speakers	NR	Random, probabilistic	1,124	≥ 35	71.3	ADA: FPG, OGTT	Primary Study
Díaz-Santana, 2014	Puerto Rico	Cross-sectional analytical	21–79 years, residents of San Juan	Incomplete data	Household complex	858	48.9*	68.6	ADA: FPG	Secondary Analysis
Cynthia Perez, 2014	Puerto Rico	Cross-sectional analytical	21–79 years, residents of San Juan	Pregnancy, inability to provide data	Three-stage cluster, probabilistic	857	49.4*	65.7	ADA: FPG, OGTT, HbA1c	Primary Study
Ramírez, 2014	Venezuela	Cross-sectional descriptive	18–70 years	< 18 or > 70 years, infectious diseases, pregnancy	Simple random, probabilistic	240	37.5*	79.2	ADA: FPG	Primary Study
Wong-McClure, 2015	Costa Rica	Cross-sectional analytical	> 20 years, non-institutionalized	Pregnancy, lactation	Stratified, multistage, probabilistic	3,653	20-≥65†	49.4	ADA: FPG	National Survey 2010
Seclen, 2015	Peru	Longitudinal	≥ 25 years, urban/suburban areas	Mental disorders, pregnancy	Multistage stratified, probabilistic	1,677	≥ 25	49.4	ADA: FPG	PERUDIAB
Kumar, 2016	Mexico	Cross-sectional analytical	> 50 years, MHAS participants	Missing HbA1c, prior diabetes	Stratified and randomized, probabilistic	2,012	62.3*	53	ADA: HbA1c	MHAS
Nieto Martinez, 2017	Venezuela	Cross-sectional analytical	> 20 years, residents > 6 months	Pregnancy, inability to communicate	Multistage stratified, probabilistic	1,334	44.8*	68.5	ADA: FPG	VEMSOLSP
Peña Cordero, 2017	Ecuador	Cross-sectional analytical	≥ 18 years, urban area of Cuenca	Pregnancy, type 2 diabetes	Stratified, probabilistic	386	38.5*	62.4	ADA: FPG, OGTT, HbA1c	Primary Study
Irazola, 2017	Argentina, Chile, Uruguay	Cross-sectional analytical	35–74 years, residents of specific cities	NR	Multistage stratified, probabilistic	7,407	35–74	58.1	WHO: FPG	CESCAS I
Orces, 2018	Ecuador	Cross-sectional analytical	≥ 60 years, residents of specific regions	NR	Multistage, probabilistic	2,298	71.5*	54.7	ADA: FPG	SABES
Souza Marques dos Santos, 2020	Brazil/UK	Cross-sectional analytical	≥ 50 years, HbA1c and data available	Missing HbA1c or data	Stage-stratified, probabilistic	7,248	61–67‡	53.2–55.1‡	ADA: HbA1c	ELSA and ELSI
Nieto-Martinez, 2020	Venezuela	Cross-sectional analytical	≥ 20 years, residents > 6 months	Pregnancy, inability to communicate	Clustered, stratified, probabilistic	3,414	41.2*	52.2	ADA: FPG, OGTT	EVESCAM
Lazo-Porras, 2020	Peru	Longitudinal	≥ 35 years, permanent residents	Pregnancy, physical disability, tuberculosis	Stratified random, probabilistic	2,345	54.3*	21.6§	ADA: FPG, HbA1c	WHO: FPG
Madsen Beau de Rochars, 2021	Dominican Republic	Cross-sectional analytical	Residents of bateyes, > 18 years	NR	Multistage cluster, probabilistic	1,293	35.0*	54.3–60.8‡	ADA: HbA1c	Primary Study
Moehlecke Iser, 2021	Brazil	Cross-sectional analytical	≥ 18 years, residents	HbA1c compatible with diabetes, missing data	Three-stage random, probabilistic	7,548	≥ 18	NR	ADA: HbA1c, WHO: HbA1c	PNS Brazil 2014–2015
Lima, 2022	Brazil	Cross-sectional analytical	Adults, urban area of Teresina	Diabetes, FPG > 126 mg	Two-stage cluster, probabilistic	224	NR	68.7	ADA: FPG	Primary Study
Basto-Abreu, 2023	Mexico	Cross-sectional analytical	≥ 20 years, blood sample	Fasting < 8 h, gestational diabetes	Multistage, stratified and clustered, probabilistic	1,945	≥ 20	NR	ADA: FPG, HbA1c	ENSANUT 2022
Fermín-Martinez, 2023	Mexico	Cross-sectional analytical	≥ 20 years, no diabetes diagnosis	Missing data, fasting < 8 h, gestational diabetes	Two-stage clustered stratified, probabilistic	21,442	44.0*	57.5	ADA: FPG, HbA1c, WHO: FPG	ENSANUT 2016–2021
Karl Peltzer, 2023	Ecuador	Cross-sectional analytical	18–69 years, no CVD	Prior CVD, pregnancy	Multistage stratified random, probabilistic	3,608	39.0*	54	ADA: FPG	STEPS 2018

†Interquartile range

ADA: American Diabetes Association; WHO: World Health Organization

FPG: Fasting Plasma Glucose; OGTT: Oral Glucose Tolerance Test; HbA1c: Glycated Hemoglobin

Regarding the demographic characteristics of participants, all studies focused on adult populations, typically including participants aged 18 or 20 years and older. However, studies such as Orces (2018) [27] in Ecuador focused exclusively on adults over 60 years old, while Kumar (2016) in Mexico studied adults over 50 years old [23] (Table 2).

**Table 2:** Bias analysis of studies on the prevalence and incidence of prediabetes in Latin America

Author and Year	1	2	3	4	5	6	7	8	9	Total
Pablo Aschner, 1992	Y	Y	Y	Y	Y	Y	Y	Y	N	8
Suely Gimeno, 1998	Y	Y	Y	Y	Y	Y	Y	Y	N	8
Torquato, 2003	Y	Y	Y	Y	Y	Y	Y	Y	N	8
Guerrero-Romero, 2008	Y	Y	Y	Y	Y	Y	Y	Y	N	8
Bosi, 2009	Y	Y	Y	Y	Y	Y	Y	Y	N	8
Jorge Escobedo, 2010	Y	Y	Y	Y	Y	Y	Y	Y	U	8
Rodriguez-Moran, 2010	Y	Y	Y	Y	Y	Y	Y	Y	N	8
Díaz-Santana, 2014	Y	Y	Y	Y	Y	Y	Y	Y	U	8
Cynthia Perez, 2014	Y	Y	Y	Y	Y	Y	Y	Y	N	8
Ramírez, 2014	Y	Y	Y	Y	Y	Y	Y	Y	N	8
Wong-McClure, 2015	Y	Y	Y	Y	Y	Y	Y	Y	U	8
Seclen, 2015	Y	Y	Y	Y	Y	Y	Y	Y	U	8
Kumar, 2016	Y	Y	Y	Y	Y	Y	Y	Y	U	8
L. Casapulla, 2017	Y	Y	Y	Y	Y	Y	Y	Y	N	8
Nieto Martinez, 2017	Y	Y	Y	Y	Y	Y	Y	Y	N	8
Peña Cordero, 2017	Y	Y	Y	Y	Y	Y	Y	Y	N	8
Irazola, 2017	Y	Y	Y	Y	Y	Y	Y	Y	N	8
Orces, 2018	Y	Y	Y	Y	Y	Y	Y	Y	N	8
Souza Marques dos Santos, 2020	Y	Y	Y	Y	Y	Y	Y	Y	N	8
Nieto-Martinez, 2020	Y	Y	Y	Y	Y	Y	Y	Y	N	8
Lazo-Porras, 2020	Y	Y	Y	Y	Y	Y	Y	Y		8
Madsen Beau de Rochars, 2021	Y	Y	Y	Y	Y	Y	Y	Y	N	8
Moehlecke Iser, 2021	Y	Y	Y	Y	Y	Y	Y	Y	N	8
Lima, 2022	Y	Y	Y	Y	Y	Y	Y	Y	N	8
Basto-Abreu, 2023	Y	Y	Y	Y	Y	Y	Y	Y	N	8
Fermín-Martinez, 2023	Y	Y	Y	Y	Y	Y	Y	Y	N	8
Karl Peltzer, 2023	Y	Y	Y	Y	Y	Y	Y	Y	N	8

Y = Yes, N = No, U = Unclear

### Bias analysis

Based on the risk of bias assessment conducted for the 25 studies included in this systematic review, it can be concluded that the overall methodological quality of the studies is high. All evaluated studies met 8 of the 9 criteria established by the assessment tool, suggesting a low risk of bias in most key methodological aspects. The studies consistently demonstrated strengths in critical areas such as selecting an appropriate sampling frame, adequate participant recruitment, sufficient sample size, detailed description of subjects and study setting, adequate coverage in data analysis, use of valid methods for prediabetes identification, standardized and reliable measurement of the condition, and application of appropriate statistical analyses.

However, it is essential to note that for all studies, the ninth criterion related to response rate and management of low response rates could not be evaluated due to a lack of specific information in the study.

### Meta-analysis of prediabetes prevalence according to ADA

The meta-analysis shows a combined prevalence of prediabetes of 24% (95% CI: 18–30%) in the Latin American populations studied [13–15, 17–25, 27–36]. Furthermore, the heterogeneity between studies is very high (I² = 100%), indicating substantial variability in prevalence estimates across different studies.

Prevalence estimates vary widely between studies, from a minimum of 6% (95% CI: 4 − 8%) in Pablo Aschner’s (1992) [13] study in Colombia to a maximum of 54% (95% CI: 52 − 56%) in Lazo-Porras’ (2020) [30] study in Peru.

Studies from different time periods showed varying prevalences. However, these variations should be interpreted with caution, as they reflect not only potential temporal changes but also differences in methodology, diagnostic criteria, and studied populations across countries and time periods.

On the other hand, notable differences are observed between countries. For instance, studies in Mexico show a wide variation (from 10 to 44%), while studies in Brazil tend to show lower prevalences (from 6 to 18%, except the ELSA study).

### Meta-analysis of prediabetes prevalence according to the WHO

The meta-analysis of prediabetes prevalence (according to WHO criteria) included five studies conducted in different Latin American countries between 1998 and 2021 [12, 16, 26, 30, 32]. Using a random-effects model, the combined prevalence of prediabetes was estimated at 11% (95% CI: 5–18%). This result suggests that, according to WHO criteria, approximately one in ten people in the studied populations could have prediabetes.

Heterogeneity between studies was very high (I² = 99%), indicating substantial variability in prevalence estimates across different studies. This heterogeneity underscores the importance of considering population-specific and study-specific factors when interpreting these results.

Prevalence estimates varied considerably between studies, from a minimum of 6% (95% CI: 6 − 8%) in Lazo-Porras’ (2020) [30] study in Peru to a maximum of 19% (95% CI: 18 − 20%) in Irazola’s (2017) [26] study conducted in argentina, chile, and uruguay. This wide variation could reflect fundamental differences in prediabetes prevalence between countries but could also be influenced by methodological differences or characteristics of the studied populations.

No clear pattern of increase or decrease in prevalence over time is observed regarding temporal trends. The oldest study (Suely Gimeno, 1998) [12] and the most recent one (Moehlecke Iser, 2021) [32] show relatively different prevalences (15% and 7%, respectively), while intermediate studies present more significant variability.

**Fig. 2:**
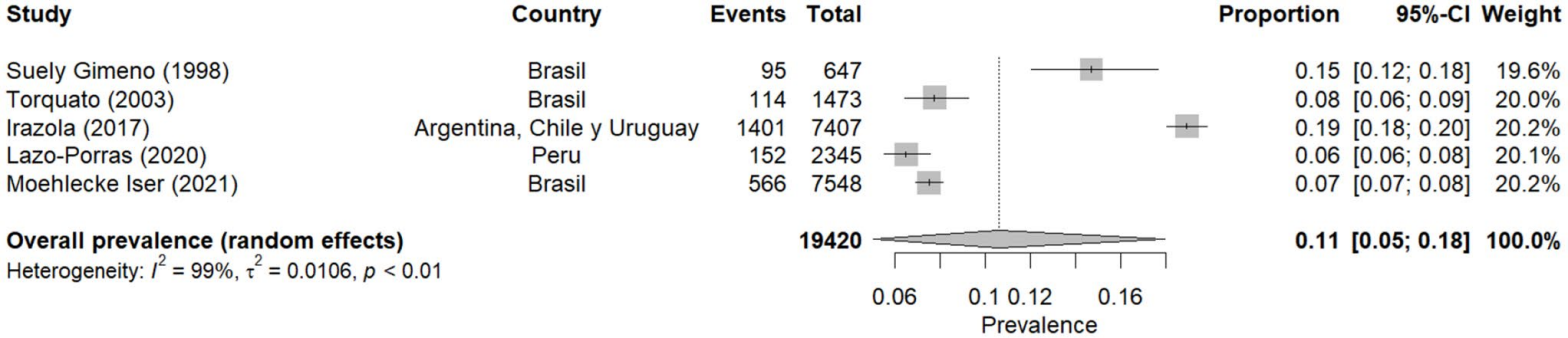
Meta-analysis of prediabetes prevalence according to WHO

Notably, three of the five studies were conducted in Brazil, showing prevalences of 15%, 8%, and 7%. This variation within the same country could reflect differences in the specific populations studied or changes in prediabetes prevalence over time in Brazil (Fig. 2).

### Meta-analysis of prediabetes prevalence according to FG

The meta-analysis of prediabetes prevalence using the FG criterion included nine studies conducted in different Latin American countries between 2010 and 2023 [18, 20–22, 24, 27, 33, 36]. Using a random-effects model, the combined prevalence of prediabetes was estimated at 18% (95% CI: 10–27%). Heterogeneity between studies was very high (I² = 99%), indicating substantial variability in prevalence estimates across different studies.

**Fig. 3:**
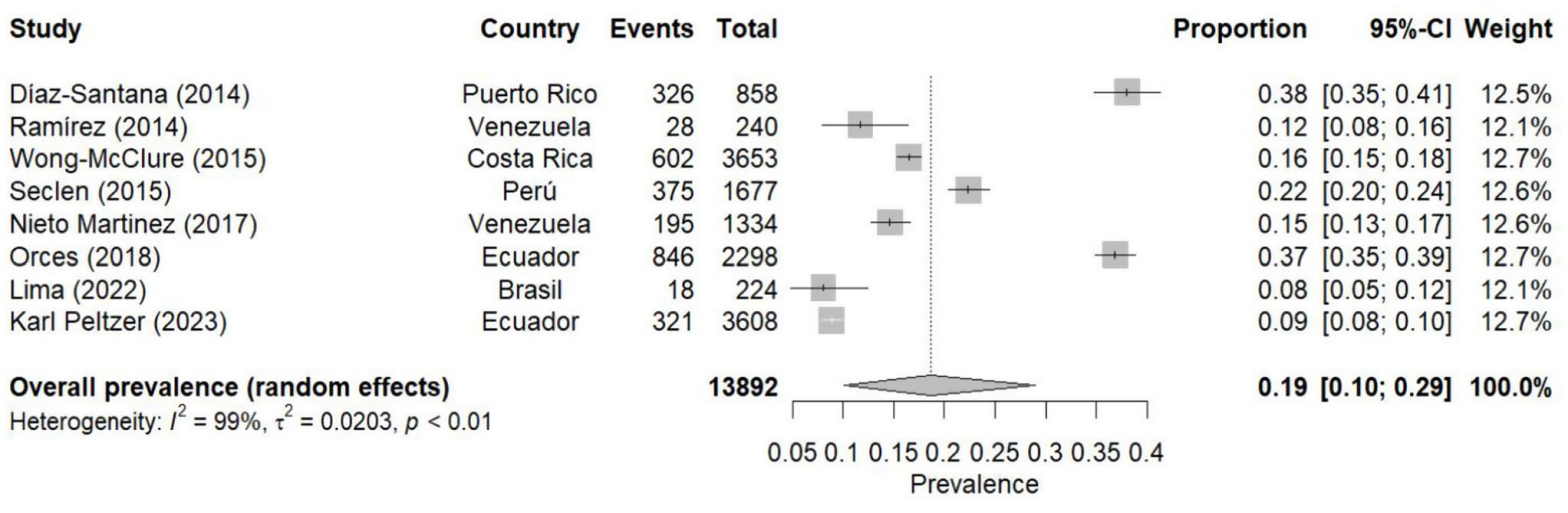
Meta-analysis of prediabetes prevalence according to fasting glucose

Prevalence estimates varied considerably between studies, from a minimum of 8% (95% CI: 5 − 12%) in Lima’s (2022) [33] study in Brazil, to a maximum of 38% (95% CI: 35 − 41%) in Díaz-Santana’s (2014) [18] study in Puerto Rico. On the other hand, regarding temporal trends, no clear pattern of increase or decrease in prevalence over time is observed. The most recent studies (Lima 2022 and Karl Peltzer 2023) [33, 36] show relatively low prevalences (8% and 9%, respectively), while some earlier studies present higher prevalences (Fig. 3).

### Meta-analysis of prediabetes prevalence according to PPG

The meta-analysis of prediabetes prevalence using the PPG criterion included five studies conducted in Latin American countries between 1992 and 2020 [13, 15, 17, 19, 29]. Using a random-effects model, the combined prevalence of prediabetes was estimated at 20% (95% CI: 3–46%). Regarding heterogeneity between studies, this was extremely high (I² = 100%).

Prevalence estimates varied considerably between studies, from a minimum of 6% (95% CI: 4 − 8%) in Pablo Aschner’s (1992) [13] study in Colombia and Bosi (2009) in Brazil [15], to a maximum of 47% (95% CI: 44 − 51%) in Cynthia Perez’s (2014) study in Puerto Rico.

**Fig. 4:**
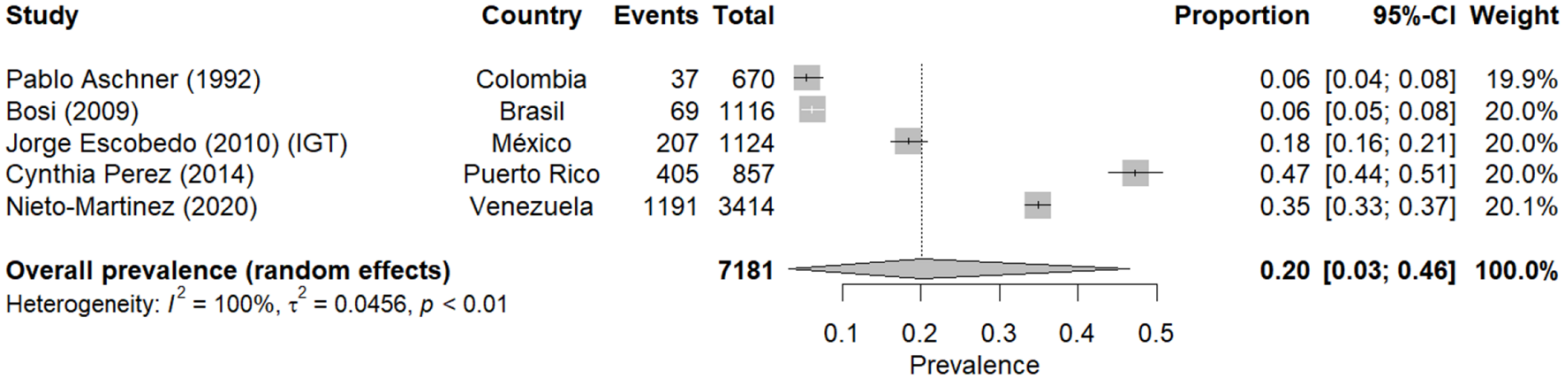
Meta-analysis of prediabetes prevalence according to postprandial glucose

A pattern of increase in prevalence over time is observed regarding temporal trends. The oldest studies (Pablo Aschner 1992 and Bosi 2009) [13, 15] show lower prevalences (6%), while more recent studies present higher prevalences, culminating with 35% in Nieto-Martinez’s (2020) [24] study in Venezuela (Fig. 4).

### Meta-analysis of prediabetes prevalence according to HbA1c

The prevalence of prediabetes using the HbA1c criterion included seven studies conducted in Latin American countries between 2016 and 2021 [23, 28, 30, 31, 36]. The combined prevalence of prediabetes, using a random-effects model, was estimated at 36% (95% CI: 21–52%). This result suggests that, according to the HbA1c criterion, approximately one in three people in the studied populations could have prediabetes.

The heterogeneity between studies was high (I² = 100%), indicating substantial variability in prevalence estimates across different studies. This heterogeneity underscores the importance of considering population-specific and study-specific factors when interpreting these results.

Prevalence estimates varied considerably between studies, from a minimum of 18% (95% CI: 18–19%) in Moehlecke’s (2017) [32] study in Ecuador, to a maximum of 54% (95% CI: 52 − 56%) in Lazo-Porras’ (2020) [28] study in Peru. On the other hand, regarding temporal trends, no clear pattern of increase or decrease in prevalence over time is observed, given that all studies were conducted in a relatively short period (2016–2021).

**Fig. 5:**
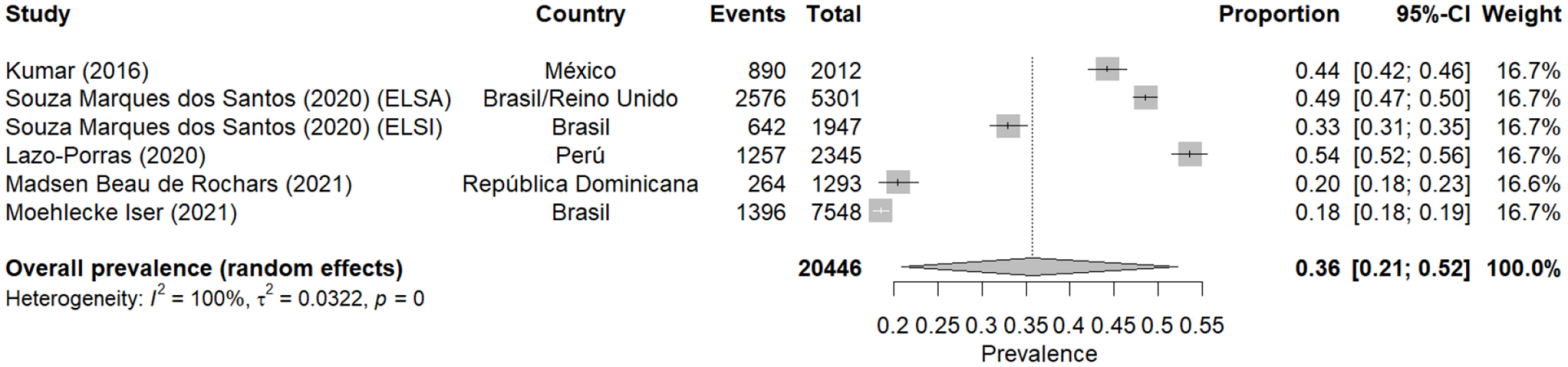
Meta-analysis of prediabetes prevalence according to glycated hemoglobin

However, notable differences are observed between countries and even within the same country. For example, in Brazil, the study by Souza Marques dos Santos (2020) [28] shows prevalences of 49% (ELSA) and 33% (ELSI), while the study by Moehlecke Iser (2021) [32] reports a prevalence of 18% (Fig. 5).

### Publication bias analysis

**Fig. 6:**
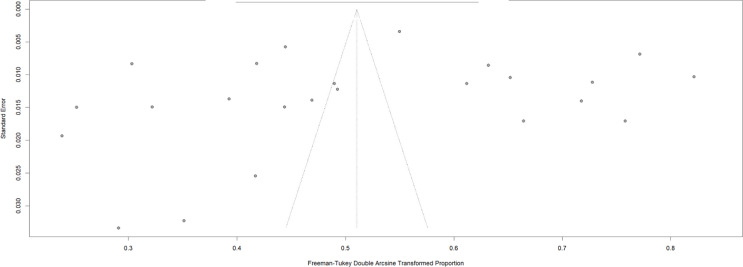
Funnel Plot of prediabetes prevalence studies

**Fig. 7:**
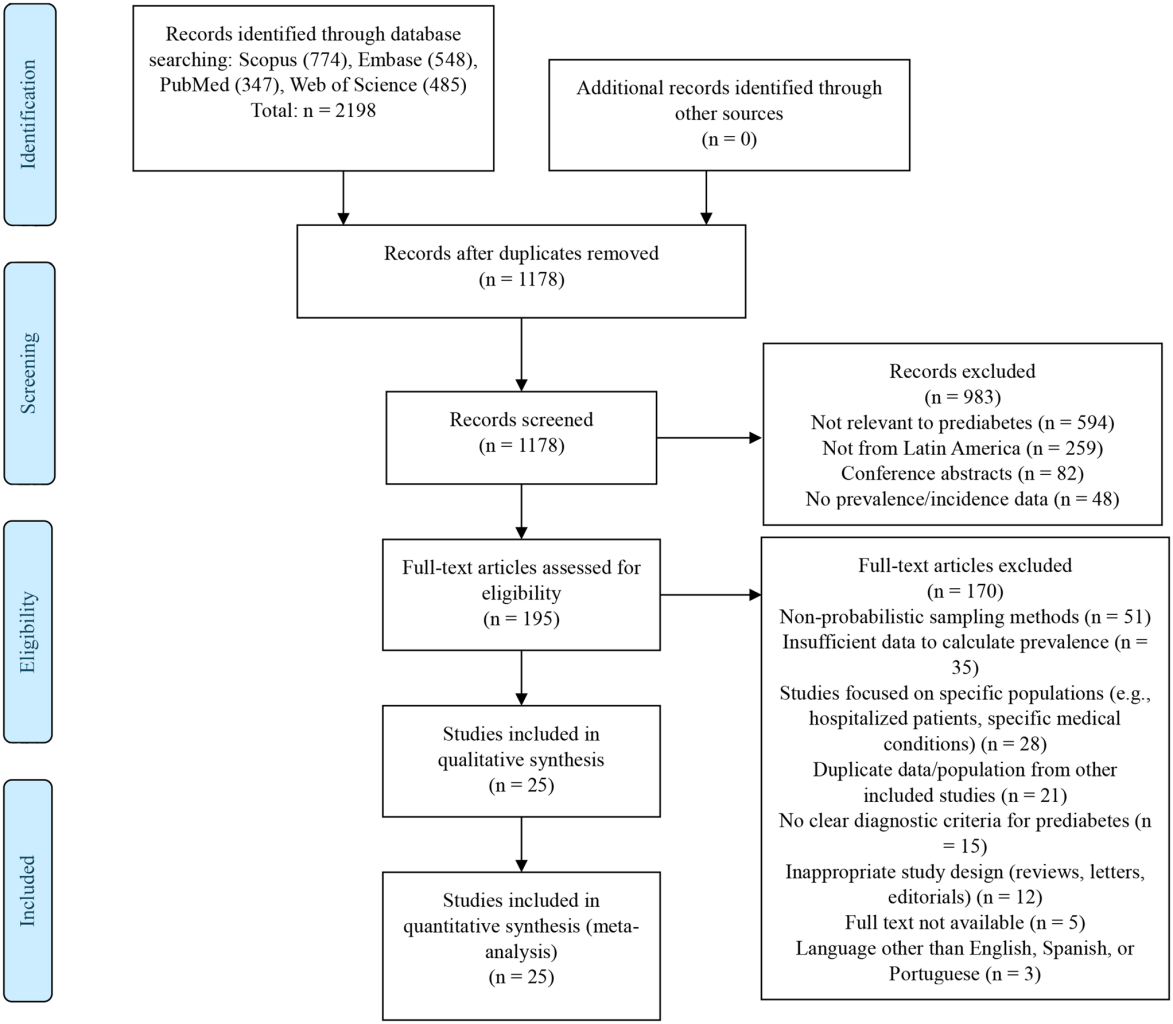
Flowchart of study selection

The funnel plot (Fig. 6) showed notable asymmetry in the distribution of studies. Egger’s regression test confirmed significant asymmetry (*p* < 0.001). The trim-and-fill analysis suggested the presence of potentially missing studies. However, the high I² values (99–100%) and significant between-study variance (τ² = 0.0638) indicated that the observed asymmetry was largely attributable to substantial heterogeneity rather than publication bias alone (Fig. 7).

## Discussion

### Interpretation of results and comparison with existing literature

This findings on the prevalence of prediabetes in Latin America generally align with previous studies conducted in the region, although they offer a broader and more updated perspective. For example, an earlier meta-analysis by Rooney et al. found that the prevalence was 11.6% (95% CI; 10.7–12.5%) [3]. This difference could be attributed to including studies with greater country diversity in our analysis, as the mentioned study only included Bolivia and Mexico. In contrast, ours included twelve countries in the region.

The findings on the prevalence of prediabetes in Latin America are comparable with previous studies, although slightly lower, to other regions of the world than those reported for some populations. For instance, in the United States, the prevalence of prediabetes has been estimated at around 38% of adults [37]. In Europe, a meta-analysis by Eades et al. in 2016 estimated a prediabetes prevalence of 22.3% using WHO criteria, which is higher than our estimate of 11% with the same criteria [38]. These comparisons suggest that, while the burden of prediabetes in Latin America is substantial, it could be lower than in some more developed regions.

This analysis includes studies spanning from 1992 to 2024, representing different periods of research in prediabetes across Latin America. While some recent studies report higher prevalences (e.g., Souza Marques dos Santos 2020 [28] and Lazo-Porras 2020 [30] reporting > 30%) compared to earlier studies (e.g., Pablo Aschner 1992 [4] and Bosi 2009 [15] reporting < 10%), these differences cannot be interpreted as direct evidence of temporal trends. This is because studies from different periods vary substantially in their methodological approaches, diagnostic criteria (particularly with the later introduction of HbA1c as a diagnostic tool), geographical locations, and studied populations. Furthermore, the evolution of screening practices and awareness of prediabetes over time may have influenced detection rates independently of true prevalence changes.

This apparent trend of increasing prediabetes prevalence over time is consistent with global observations. The International Diabetes Federation (IDF) has reported a steady increase in the prevalence of diabetes and prediabetes worldwide, attributing this increase to factors such as urbanization, population aging, and changes in lifestyles towards less healthy diets and increased sedentarism [39]. Our findings suggest that Latin America is not an exception to this global trend.

However, it is important to note that interpreting these temporal trends should be done cautiously. The observed differences between older and more recent studies could be due not only to fundamental changes in prediabetes prevalence but also to improvements in detection methods, changes in diagnostic criteria (such as the introduction of HbA1c as a diagnostic criterion) [9], and increased awareness about the importance of prediabetes that could have led to an increase in detection efforts. Future longitudinal and trend studies will be crucial to determine more precisely how the prevalence of prediabetes is evolving in Latin America over time.

#### Prediabetes prevalence according to different diagnostic criteria

The observed differences in prediabetes prevalence across diagnostic criteria (WHO criteria, fasting glucose, postprandial glucose, and HbA1c) are notable and deserve detailed discussion. Our meta-analysis revealed substantial variations: WHO criteria showed the lowest prevalence at 11% (95% CI: 5–18%), followed by fasting glucose at 18% (95% CI: 10–27%), postprandial glucose at 20% (95% CI: 3–46%), and HbA1c showing the highest at 32% (95% CI: 21–52%).

These discrepancies can be attributed to the inherent characteristics of each evaluation method. While fasting glucose focuses on fasting alterations, postprandial glucose examines the metabolic response to a glucose load, potentially identifying anomalies that might go unnoticed during fasting. HbA1c, on the other hand, offers a perspective of average glycemic control over a more extended period, typically two to three months.

These findings emphasize the relevance of employing multiple diagnostic criteria to comprehensively assess prediabetes, particularly in heterogeneous populations such as Latin America. The selection of the most appropriate diagnostic method may vary according to the specific characteristics of the population and the clinical context in question. Additionally, it underscores the need to consider multiple diagnostic criteria for a comprehensive diabetes risk assessment, especially in populations where factors such as diet, lifestyle, and genetic characteristics can influence the manifestation of dysglycemia [40].

### Prediabetes prevalence in the context of latin American public health

The prevalence of prediabetes found in our meta-analysis has profound implications for public health in Latin America. This figure is alarming and underscores the magnitude of the challenge faced by Latin American health systems in the prevention and management of chronic non-communicable diseases [41].

In the context of limited health resources in many Latin American countries, this high prevalence of prediabetes represents a significant potential burden. If not adequately addressed, it could translate into a substantial increase in type 2 diabetes cases in the coming decades, with the consequent associated micro and macrovascular complications. This would affect the quality of life of millions of people and exert considerable pressure on the already overburdened health systems in the region [42].

The variability in prevalences observed among countries and subregions of Latin America highlights the need for public health strategies adapted to specific local contexts. Factors such as urbanization, changes in dietary patterns, sedentarism, and socioeconomic disparities, which vary significantly within the region, likely contribute to these differences and should be considered in the design of interventions [43].

From an economic perspective, the high prevalence of this metabolic alteration represents a crucial opportunity for prevention. Studies have shown that lifestyle interventions and, in some cases, pharmacological interventions in individuals with prediabetes can prevent or delay progression to T2DM [7]. Given that the cost of managing diabetes and its complications is substantially higher than that of preventive interventions, investing in early detection and management programs for prediabetes could result in significant long-term savings for Latin American health systems [44].

The high prevalence of prediabetes also underscores the importance of a “health in all policies” approach in Latin America. Addressing this problem requires interventions from the health sector and intersectoral policies that promote healthier environments, improve access to nutritious foods, encourage physical activity, and address the social determinants of health. Successful experiences in some countries of the region, such as taxes on sugar-sweetened beverages in Mexico, demonstrate the potential of these policies to influence risk factors for prediabetes and diabetes [45].

Finally, our findings highlight the urgent need to improve epidemiological surveillance systems in Latin America. The variability in diagnostic methods and the lack of consistent data in some countries of the region hinder a precise understanding of the burden of prediabetes. Strengthening these systems would allow for more effective monitoring of temporal and geographical trends, facilitating the evaluation of the impact of public health interventions and the efficient allocation of resources.

### Strengths and limitations of the study

This meta-analysis presents several strengths: broad geographical coverage encompassing 9 Latin American countries, extensive temporal coverage spanning three decades (1992–2024), inclusion of multiple diagnostic criteria for prediabetes, use of robust statistical methods including random-effects models, strict inclusion of only probabilistic sampling studies, comprehensive assessment of study quality and risk of bias, and separate analysis of different diagnostic criteria.

However, the study faces important limitations. The high heterogeneity observed among studies (I² = 99–100%) suggests substantial variability that methodological or population differences cannot fully explain. The evidence of possible publication bias observed in the funnel plot advises caution in interpreting the combined results. Additional limitations include: unequal representation of Latin American countries, variable sampling methods across studies, differences in operational definitions of prediabetes, limited number of incidence studies, lack of data from rural populations in many countries, and variable reporting of population characteristics across studies.

Another key limitation of this review is the inability to establish clear temporal trends in prediabetes prevalence across Latin America. While the included studies span several decades, the heterogeneity in methodological approaches, diagnostic criteria, and geographical coverage makes direct temporal comparisons inappropriate. Future longitudinal studies within specific countries, using consistent diagnostic criteria and methodologies, would be necessary to properly assess temporal changes in prediabetes prevalence.

Nevertheless, despite these limitations, this meta-analysis provides valuable insights into prediabetes prevalence in Latin America.

## Conclusions

This systematic review provides evidence of a high prevalence of prediabetes in Latin America, with a combined estimate of 24% (95% CI: 18–30%). The results reveal significant variability in prevalences according to the diagnostic criteria used and among different countries in the region. The prevalence based on ADA criteria was higher than that of WHO, while the prevalence based on HbA1c was notably higher than PPG and FG. These differences underscore the crucial importance of standardization in diagnostic methods. Meanwhile, the observed heterogeneity among studies reflects the diversity of Latin American populations and different methodological approaches, highlighting the need for contextualized interpretations.

Based on the results of this meta-analysis, we recommend implementing comprehensive public health strategies adapted to the local context to address the high prevalence of prediabetes in Latin America. These strategies should include (1) Standardization of diagnostic criteria and prediabetes detection methods throughout the region, considering the ethnic and demographic particularities of each population. (2) Strengthening epidemiological surveillance systems to monitor prediabetes trends and evaluate the impact of interventions. (3) Implement evidence-based prevention programs focused on lifestyle modifications, particularly in high-risk populations. (4) Development of intersectoral policies that address the social determinants of health and promote environments that facilitate healthy choices. (5) Investment in additional research, especially longitudinal studies using consistent methodology within countries, to better understand temporal trends in prediabetes prevalence in the Latin American context. (6) Training of health personnel in early detection and management of prediabetes. (7) Public awareness about the importance of prediabetes and prevention strategies. These recommendations seek not only to reduce the burden of prediabetes but also to mitigate the future impact of type 2 diabetes on Latin American health systems and societies.

Looking forward, we identify several key areas for improvement in future research and practice. First, there is a critical need for standardization of diagnostic criteria and detection methods throughout the region, considering the ethnic and demographic particularities of each population. Second, strengthening epidemiological surveillance systems is essential to better monitor prediabetes trends and evaluate intervention impacts. Third, future research should prioritize longitudinal studies, as our review found only one such study, limiting our understanding of incidence rates and disease progression in Latin American populations. Fourth, there is a need to expand research to underrepresented countries and rural areas to provide a more comprehensive picture of prediabetes in the region. Fifth, the development of standardized protocols for screening and detection would improve data comparability across studies. Finally, the implementation of culturally appropriate interventions and cost-effective screening strategies should be prioritized to address the growing burden of prediabetes in Latin America.

## Electronic supplementary material

Below is the link to the electronic supplementary material.

Supplementary Material 1

## Data Availability

Data are available upon request to the corresponding author.
