# Supplementary material for: Prevalence and incidence of prediabetes in Latin America. A systematic review and meta-analysis
Source: J Diabetes Metab Disord. 2024 Dec 27;24(1):25. doi: 10.1007/s40200-024-01549-6 (PMC11680525; doi:10.1007/s40200-024-01549-6)
Supplement: Supplementary file 1 — Supplementary Material 1 [file 40200_2024_1549_MOESM1_ESM.docx]

Supplementary file 1

| Search strategy in PUBMED | |
| --- | --- |
| #1 | prediabetes[TIAB] OR "pre-diabetes"[TIAB] OR hyperglycemia[TIAB] OR "hyperglycaemia"[TIAB] OR "impaired fasting glucose"[TIAB] OR "glucose intolerance"[TIAB] OR "impaired glucose tolerance"[TIAB] OR "impaired fasting glycaemia"[TIAB]) AND ("prevalence"[TIAB] OR "incidence"[TIAB] |
| #2 | "Antigua and Barbuda"[TIAB] OR "Antigua y Barbuda"[TIAB] OR "Argentina"[TIAB] OR "Bahamas"[TIAB] OR "Barbados"[TIAB] OR "Belize"[TIAB] OR "Bolivia"[TIAB] OR "Brazil"[TIAB] OR "Brasil"[TIAB] OR "United States Virgin Islands"[TIAB] OR "British Virgin Islands"[TIAB] OR "Chile"[TIAB] OR "Colombia"[TIAB] OR "Costa Rica"[TIAB] OR "Cuba"[TIAB] OR "Dominica"[TIAB] OR "Dominican Republic"[TIAB] OR "República Dominicana"[TIAB] OR "Ecuador"[TIAB] OR "El Salvador"[TIAB] OR "Grenada"[TIAB] OR "Granada"[TIAB] OR "Guatemala"[TIAB] OR "Guyana"[TIAB] OR "Haiti"[TIAB] OR "Haïti"[TIAB] OR "Honduras"[TIAB] OR "Jamaica"[TIAB] OR "Mexico"[TIAB] OR "México"[TIAB] OR "Nicaragua"[TIAB] OR "Panama"[TIAB] OR "Panamá"[TIAB] OR "Paraguay"[TIAB] OR "Peru"[TIAB] OR "Perú"[TIAB] OR "Puerto Rico"[TIAB] OR "Saint Kitts and Nevis"[TIAB] OR "San Cristóbal y Nieves"[TIAB] OR "San Cristobal y Nieves"[TIAB] OR "Saint Lucia"[TIAB] OR "Santa Lucía"[TIAB] OR "Saint Vincent and the Grenadines"[TIAB] OR "San Vicente y las Granadinas"[TIAB] OR "Suriname"[TIAB] OR "Surinam"[TIAB] OR "Trinidad and Tobago"[TIAB] OR "Trinidad y Tobago"[TIAB] OR "West Indies"[TIAB] OR "Indias occidentales"[TIAB] OR "Uruguay"[TIAB] OR "Venezuela"[TIAB] OR "Latin America"[TIAB] OR "latinoamérica"[TIAB] OR "latin amer*"[TIAB] OR "South America"[TIAB] OR "south amer*"[TIAB] OR "Central America"[TIAB] OR "central amer*"[TIAB] |
| #3 | #1 AND #2 |
| Search strategy in EMBASE | |
| #1 | ('prediabetes'/exp OR 'pre-diabetes' OR hyperglycemia OR hyperglycaemia OR 'impaired fasting glucose' OR 'glucose intolerance' OR 'impaired glucose tolerance' OR 'impaired fasting glycaemia') AND ('prevalence'/exp OR 'incidence'/exp) |
| #2 | ('antigua and barbuda':ti,ab,kw OR 'antigua y barbuda':ti,ab,kw OR 'argentina':ti,ab,kw OR 'bahamas':ti,ab,kw OR 'barbados':ti,ab,kw OR 'belize':ti,ab,kw OR 'bolivia':ti,ab,kw OR 'brazil':ti,ab,kw OR 'brasil':ti,ab,kw OR 'united states virgin islands':ti,ab,kw OR 'british virgin islands':ti,ab,kw OR 'chile':ti,ab,kw OR 'colombia':ti,ab,kw OR 'costa rica':ti,ab,kw OR 'cuba':ti,ab,kw OR 'dominica':ti,ab,kw OR 'dominican republic':ti,ab,kw OR 'república dominicana':ti,ab,kw OR 'ecuador':ti,ab,kw OR 'el salvador':ti,ab,kw OR 'grenada':ti,ab,kw OR 'granada':ti,ab,kw OR 'guatemala':ti,ab,kw OR 'guyana':ti,ab,kw OR 'haiti':ti,ab,kw OR 'haïti':ti,ab,kw OR 'honduras':ti,ab,kw OR 'jamaica':ti,ab,kw OR 'mexico':ti,ab,kw OR 'méxico':ti,ab,kw OR 'nicaragua':ti,ab,kw OR 'panama':ti,ab,kw OR 'panamá':ti,ab,kw OR 'paraguay':ti,ab,kw OR 'peru':ti,ab,kw OR 'perú':ti,ab,kw OR 'puerto rico':ti,ab,kw OR 'saint kitts and nevis':ti,ab,kw OR 'san cristóbal y nieves':ti,ab,kw OR 'san cristobal y nieves':ti,ab,kw OR 'saint lucia':ti,ab,kw OR 'santa lucía':ti,ab,kw OR 'saint vincent and the grenadines':ti,ab,kw OR 'san vicente y las granadinas':ti,ab,kw OR 'suriname':ti,ab,kw OR 'surinam':ti,ab,kw OR 'trinidad and tobago':ti,ab,kw OR 'trinidad y tobago':ti,ab,kw OR 'west indies':ti,ab,kw OR 'indias occidentales':ti,ab,kw OR 'uruguay':ti,ab,kw OR 'venezuela':ti,ab,kw OR 'latin america':ti,ab,kw OR 'latinoamérica':ti,ab,kw OR 'latin amer$':ti,ab,kw OR 'south america':ti,ab,kw OR 'south amer$':ti,ab,kw OR 'central america':ti,ab,kw OR 'central amer$':ti,ab,kw) |
| #3 | #1 AND #2 |
| Search strategy in Scopus | |
| #1 | TITLE-ABS-KEY ( "prediabetes" OR "pre-diabetes" OR hyperglycemia OR "hyperglycaemia" OR "impaired fasting glucose" OR "glucose intolerance" OR "impaired glucose tolerance" OR "impaired fasting glycaemia" ) |
| #2 | TITLE-ABS-KEY ( "prevalence" OR "incidence" ) AND TITLE-ABS-KEY ( "Antigua and Barbuda" OR "Antigua y Barbuda" OR "Argentina" OR "Bahamas" OR "Barbados" OR "Belize" OR "Bolivia" OR "Brazil" OR "Brasil" OR "United States Virgin Islands" OR "British Virgin Islands" OR "Chile" OR "Colombia" OR "Costa Rica" OR "Cuba" OR "Dominica" OR "Dominican Republic" OR "República Dominicana" OR "Ecuador" OR "El Salvador" OR "Grenada" OR "Granada" OR "Guatemala" OR "Guyana" OR "Haiti" OR "Haíti" OR "Honduras" OR "Jamaica" OR "Mexico" OR "México" OR "Nicaragua" OR "Panama" OR "Panamá;" OR "Paraguay" OR "Peru" OR "Perú;" OR "Puerto Rico" OR "Saint Kitts and Nevis" OR "San Cristóbal y Nieves" OR "San Cristobal y Nieves" OR "Saint Lucia" OR "Santa Lucía" OR "Saint Vincent and the Grenadines" OR "San Vicente y las Granadinas" OR "Suriname" OR "Surinam" OR "Trinidad and Tobago" OR "Trinidad y Tobago" OR "West Indies" OR "Indias occidentales" OR "Uruguay" OR "Venezuela" OR "Latin America" OR "latinoamérica" OR "latin amer$" OR "South America" OR "south amer$" OR "Central America" OR "central amer$" ) |
| #3 | #1 AND #2 |
| Search strategy in Web of Science | |
| #1 | TS=(prediabetes OR "pre-diabetes" OR hyperglycemia OR hyperglycaemia OR "impaired fasting glucose" OR "glucose intolerance" OR "impaired glucose tolerance" OR "impaired fasting glycaemia") |
| #2 | TS=("Antigua and Barbuda" OR "Antigua y Barbuda" OR Argentina OR Bahamas OR Barbados OR Belize OR Bolivia OR Brazil OR Brasil OR "United States Virgin Islands" OR "British Virgin Islands" OR Chile OR Colombia OR "Costa Rica" OR Cuba OR Dominica OR "Dominican Republic" OR "República Dominicana" OR Ecuador OR "El Salvador" OR Grenada OR Granada OR Guatemala OR Guyana OR Haiti OR Haíti OR Honduras OR Jamaica OR Mexico OR México OR Nicaragua OR Panama OR Panamá OR Paraguay OR Peru OR Perú OR "Puerto Rico" OR "Saint Kitts and Nevis" OR "San Cristóbal y Nieves" OR "San Cristobal y Nieves" OR "Saint Lucia" OR "Santa Lucía" OR "Saint Vincent and the Grenadines" OR "San Vicente y las Granadinas" OR Suriname OR Surinam OR "Trinidad and Tobago" OR "Trinidad y Tobago" OR "West Indies" OR "Indias occidentales" OR Uruguay OR Venezuela OR "Latin America" OR latinoamérica OR "latin amer*" OR "South America" OR "south amer*" OR "Central America" OR "central amer*") |
| #3 | #1 AND #2 |
